# The high photocatalytic efficiency and stability of LaNiO_3_/g-C_3_N_4_ heterojunction nanocomposites for photocatalytic water splitting to hydrogen

**DOI:** 10.1186/s13065-020-00719-w

**Published:** 2020-10-29

**Authors:** Changyu Ye, Rui Wang, Haoyu Wang, Fubin Jiang

**Affiliations:** Department of Chemistry, Beijing Normal University, Beijing, 100875 China

**Keywords:** LaNiO_3_, Polymeric graphitic carbon nitride, Z-scheme heterostructure, Photocatalysis, Hydrogen production

## Abstract

A binary direct Z-scheme LaNiO_3_/g-C_3_N_4_ nanocomposite photocatalyst consisted with LaNiO_3_ nanoparticles and g-C_3_N_4_ nanosheets was successfully synthesized by means of mechanical mixing and solvothermal methods in order to improve the photocatalytic water splitting activity. The as-prepared materials were characterized by powder X-ray diffraction (XRD), Scanning Electron microscope (SEM), Transmission Electron microscope (TEM), X-ray photoelectron spectroscope (XPS), Fourier Transform Infrared Spectroscopy (FT-IR) and N_2_ adsorption–desorption experiments, respectively, demonstrating the formation of interfacial interaction and heterogeneous structure in LaNiO_3_/g-C_3_N_4_ nanocomposites. Under UV-light irradiation, the LaNiO_3_/g-C_3_N_4_ samples which without the addition of any noble metal as co-catalyst behaved enhanced photocatalytic water splitting activity compared with pure LaNiO_3_ and g-C_3_N_4_, owing to the Z-scheme charge carrier transfer pathway. Especially, the LaNiO_3_/70%g-C_3_N_4_ nanocomposite reach an optimal yield of up to 3392.50 µmol g^−1^ in 5 h and held a maximum H_2_ evolution rate of 678.5 µmol h^−1^ g^−1^ that was 5 times higher than that of pure LaNiO_3_. 
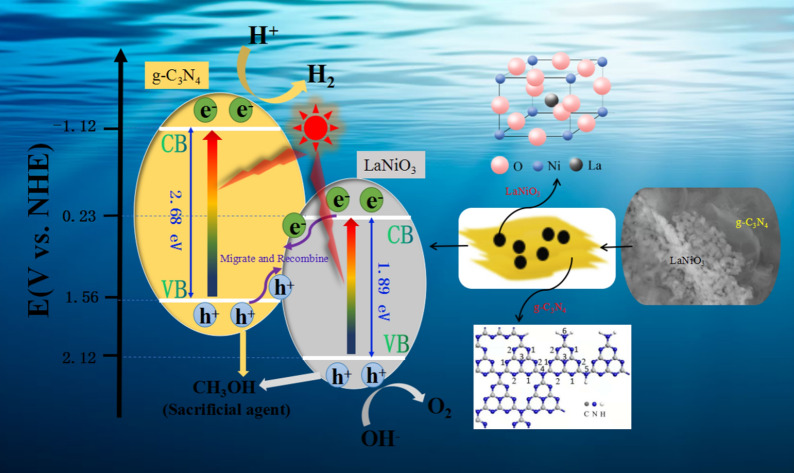

## Introduction

With the rapid development of modern society, there has been appeared the exhaustion of fossil fuels and environmental deterioration crisis. One form of environmentally friendly, economical and low cost renewable alternative energy was urgently needed under recent situation. Because of the high energy value and the benefit of green cleaning, hydrogen (H_2_) is a kind of the promising alternative new energy source that can become the substitute of the traditional carbon-based fossil fuels [1–4]. The light-driven photocatalytic water splitting to produce hydrogen has become the most promising method for scientific research to obtain sustainable energy [5, 6]. Researchers from all the world had found a mount of semiconductor photocatalyst showing good photocatalytic activities for water splitting to produce hydrogen (H_2_) [7–9]. Perovskite-type semiconductor materials present excellent photocatalytic properties due to sufficient oxygen vacancies and variable metal valence. However, there still exist some apparent disadvantages about these catalytic material such as poor light stability, high electron–hole recombination rate and small surface area that contribute to the reduction of catalytic activity and stability [10–14]. Several approaches have been proposed to overcome these problems, such as doping metal (Cu, Al) [15–18] or metal compound (CdS, NiS, TiO_2_, BiVO_4_) [19–23] even non-metal (B, C, N) [24, 25], loading noble metal (Au, Pd, Pt) [26] for surface modification. All above measures could effectively promote the separation rate of photo-generated electrons and holes, thus improve the catalytic activities. However, metals, especially precious metals, do not meet the current low cost requirement, and the catalytic effect is still limited.

In addition, several perovskite-type heterojuction nanocomposites have already been proposed and prepared for photocatalytic reaction in the literature [27–29]. The perovskite-type materials could be combined with other metal-free semiconductor materials that have matching band gaps to form Z-scheme heterostructure photocatalytic composite materials, which can transfer the photogenerated holes or electros from one semiconductor to another, thereby they not only reduce the recombination of carriers but also acquire more wide range of light response areas [30–32]. Acharya et al. loaded LaFeO_3_ nanoparticles on RGO nanosphere, which promoted the H_2_ evolution rate more than two fold than neat LFO [33]. Similarly, Tao Lv et al. reported that graphene-encapusulated LaNiO_3_ nanoreactor perform high photocatalytic activity for water splitting that was 12 times higher than that of pure LaNiO_3_ [34]. Even though the joint of ABO_3_ type perovskite and RGO was good for the photocatalytic reaction, complicated preparation and expensive cost limited its widely application. Compared with RGO, polymeric graphitic carbon nitride (g-C_3_N_4_) as one of the best π-conjugated carbonaceous materials, also having larger surface area, high thermochemical stability, excellent electronic properties and suitable forbidden band width (2.7 eV) [35–39]. More importantly, simple preparation process and cheap raw materials made g-C_3_N_4_ become a promising and popular material for photocatalytic reactions, as we can found, Fe_2_O_3_/g-C_3_N_4_, MoS_2_/g-C_3_N_4_, CsPbBr_3_/g-C_3_N_4_, BaTiO_3_/g-C_3_N_4_ [40–43] and so on materials had already been reported in the past few years. Ke Xu et al. combined g-C_3_N_4_ with LaFeO_3_ to prepare a heterogeneous composite material [44], which slightly improved the catalytic activity for water splitting compared to pure LaFeO_3_. Xiaosong Zhou et al. successfully synthesis LaNiO_3_/g-C_3_N_4_ for enhancing visible light photocatalytic activity towards tetracycline degradation [45], and the TC degradation rate was about 3.8 and 3.9 times larger than those of pure g-C_3_N_4_ and pristine LaNiO_3_, respectively. LaNiO_3_ synthesized via a sol–gel had been investigated for photocatalytic H_2_ evolution and the degradation of organic matter [46, 47], but the effect still needed to improve. As far as we know, it had not yet reported that LaNiO_3_ and g-C_3_N_4_ based Z-type heterogeneous structure catalysts can effectively utilize solar energy to catalytic decomposition of water, so it is necessary to do further research for the establishment of the catalyst system.

In this paper, Z-scheme-based photocatalytic system LaNiO_3_/x %g-C_3_N_4_ were constructed by mechanical mixing and solvothermal methods. During the preparation process, LaNiO_3_ nanoparticles prepared by sol–gel method were evenly loaded on g-C_3_N_4_ layered nanosheets to obtain different products with a series of doping ratios. Finally, water splitting experiments showed that catalyst loaded 70wt % g-C_3_N_4_ present the best photocatalytic activity and perform good photo stability for H_2_ evolution after five photocatalytic cycles in 20 h.

## Experimental

### Materials

Lanthanum(III) nitrate hexahydrate (purity > 99.99%), nicke(II)nitrate hexahydrate (purity > 99.99%), citric acid (purity > 99%), urea (purity > 99%), and ammonia solution(25% ~ 28%) bought from Beijing Hawk Science & Technology Co., Ltd. All other reagents used in this study were analytically pure and used without further purification.

### Synthesis of LaNiO_3_ (LNO)

LaNiO_3_ nanoparticles were synthesized by a sol–gel method. The calculated amount of La(NO_3_)_3_·6H_2_O, Ni(NO_3_)_2_·9H_2_O, and citric acid in the molar ratio of 1:1:2 was suspended in 10 ml deionized water under magnetic stirring. Then the pH value of the mixed solution was adjusted to about 1 by the addition of ammonia solution. The mixture was continuously and uniformly stirred at the temperature of 70 °C until it formed green nitrate–citrate sol. Then the gel was evaporated at 120 °C in a blast drying oven to generate bulk dry gel. Finally, the dry gel was calcined in a muffle furnace at 400 °C for 2 h and then raised the temperature to 700 °C for 4 h to obtain LaNiO_3_ powder.

### Synthesis of g-C_3_N_4_ (CN)

Graphitic carbon nitride was prepared from urea using a thermal condensation method. First, a certain amount of urea was dried at 80 °C in a blast drying oven for 24 h and grinded into fine powder. Then urea was placed in an alumina crucible with a cover and then subjected to calcination at 550 °C for 3 h in a temperature programmed muffle furnace. The ramping rate was set to 5 °C/min. Finally, the product was naturally cooled to room temperature and grinded with a mortar. And g-C_3_N_4_ yellow powder was obtained.

### Synthesis of the LaNiO_3_/g-C_3_N_4_ nanocomposite (LNCN)

**Fig. 1:**
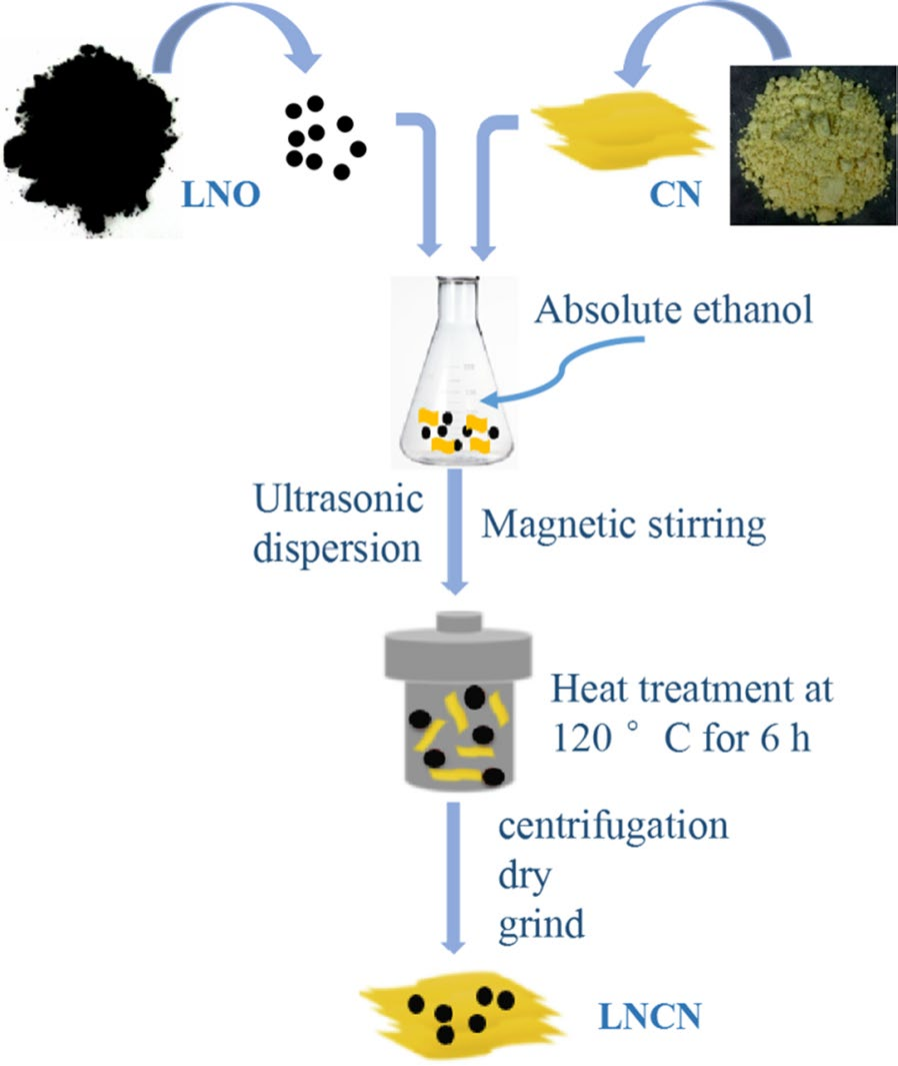
The fabrication procedure of LNCN nanocomposite

LNCN nanocomposite was synthesized by facile ultrasonication strategy, continuous stirring process and solvothermal method. The specific procedure of fabricating LNCN is displayed in Fig. 1. Firstly, stoichiometric amount of LaNiO_3_ and g-C_3_N_4_ was separately suspended into double absolute ethanol under ultra-sonication for 30 min. Then, LaNiO_3_ suspension was slowly added to ethanol contented g-C_3_N_4_ under stirred and again ultra-sonicated for 30 min. After that, the mixed solution was stirring at environment temperature for 12 h and transfered to solvothermal kettle to heat at 120 °C for 6 h. At last, the mixture was centrifuged and dried overnight at 80 °C. The samples containing different g-C_3_N_4_ to LaNiO_3_/g-C_3_N_4_ contents (20, 30, 40, 50, 60, 70, 80 and 90wt %) were labelled as LNCN2 ~ LNCN9, respectively.

### Characterization

Powder X-ray diffraction (XRD) was tested on a Shimadzu Maxima X-ray diffractometer (XRD 7000) with Cu Kα radiation at a current of 30 mA and a voltage of 40 kV. The textural structures and surface morphologies of obtained samples were analyzed by a Scanning Electron microscope (SEM, HITACHI S-4800) and TEM images were performed on a FEI Talos F200S Transmission Electron microscope. X-ray photoelectron spectroscopy (XPS) was carried out using a ThermoFisher electron spectrometer (ESCSLAB 250Xi) with a monochromatized microfocused Al X-ray source. The binding energy was calibrated using C1s peak at 284.6 eV as the reference energy. Fourier Transform Infrared Spectroscopy (FTIR) was tested on a Shimadzu IRAffinity-1 using K-Br plate applied for observation of further structural information in the range of 4000 ~ 500 cm^−1^. The specific surface area, pore volume and pore size distribution of prepared photocatalysts were measured using a Quantachrome instrument and calculated by the nitrogen adsorption–desorption isotherms using the Brunauer–Emmett–Teller (BET) and Barrett-Joyner-Halenda (BJH) methods at liquid nitrogen temperature. The evolution amount of H_2_ was detected on a Shimadzu gas chromatograph (GC-2014C) by manual injection.

### Photocatalytic experiment

Photocatalytic hydrogen production experiment was conducted by a homemade quartz reactor containing 0.03 g as-prepared catalyst suspended in 30 ml of 10 vol % CH_3_OH (aq). Methanol was used as a hole scavenger and without adding any noble metal as co-catalyst. After fully dispersed by using ultrasonic dispersion, the mixture solution was bubbled with high purity N_2_ for 30 min to remove the dissolved oxygen. A 300 W Xenon lamp light (PLS-SXE300) was used as the UV-light source (250 < λ < 380 nm) to irradiate the quartz reactor for several hours and the reaction kept at room temperature. The gas chromatograph equipped with a molecular sieve column and TCD detector was used to monitor the H_2_ evolution rate every hour and we toke manual injection method using a gas injector. During the experiment, in order to prohibit particle settlement at the bottom of the reactor, the solution was kept under constant stirring with a magnetic stirrer. It was worth mentioning that we all over took three repeated trials for every sample in order to ensure the reliability of as-obtained data. The photocatalytic stability and recyclability was tested for LNCN7 within 20 h, and every four hours was a cycle.

## Results and discussions

### XRD spectra analysis

**Fig. 2:**
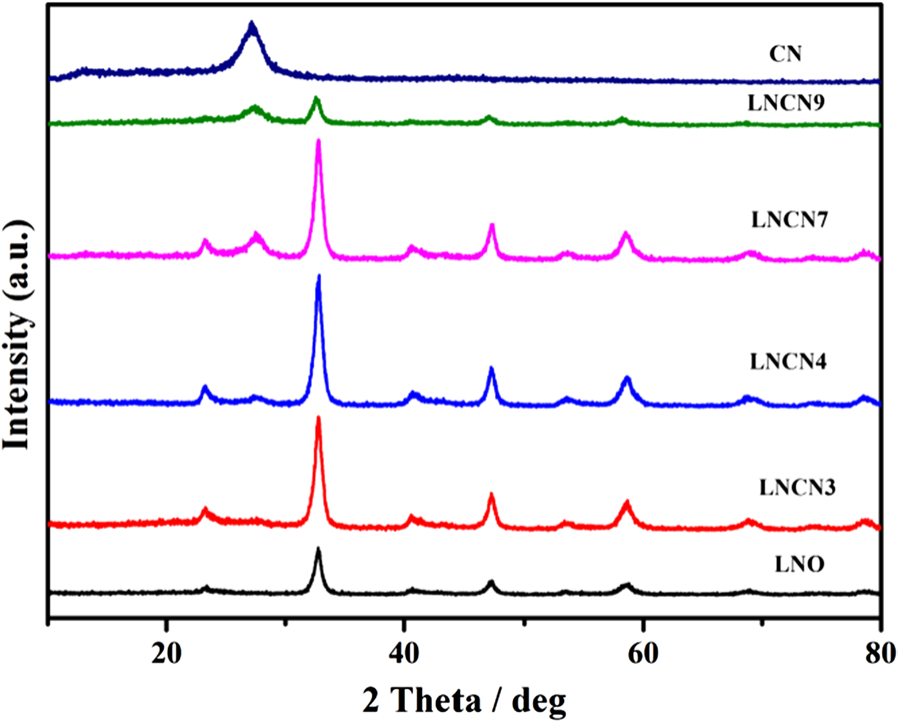
XRD patterns of pure g-C_3_N_4_, pure LaNiO_3_ and series of LNCN over the 2θ range of 10–80°

Figure 2 present the XRD patterns of pristine g-C_3_N_4_, LaNiO_3_ and LaNiO_3_/g-C_3_N_4_ heterojunction nanocomposites with various doping ratios of g-C_3_N_4_. It could be seen g-C_3_N_4_ had one remarkable diffraction peak at 2θ = 27.2°, which could be assigned to the (0 0 2) facet of g-C_3_N_4_. There were a number of diffraction peaks of pure LaNiO_3_ at 23.3°, 32.9°, 40.7°, 47.4°, 53.8°, 58.8°, 68.8° and 78.9°, corresponding well to (1 0 1), (1 1 0), (0 2 1), (2 0 2), (2 1 1), (1 2 2), (2 2 0) and (3 1 2) crystal planes of perovskite-type LaNiO_3_ (JCPDS Card No. 34-1181), respectively, indicating the high crystallinity of LaNiO_3_ prepared by sol–gel method [48]. For the series LNCN nanocomposites samples, the diffraction peaks basically retained the characteristic of pure LaNiO_3_ and g-C_3_N_4_. What’s more, peaks around at 27.2° were gradually increased with the increase of the doping ratio of g-C_3_N_4_ in the samples. The crystallite size can be calculated by the following Scherrer formula:

1 $$ Dp\;\text{ = }\;\frac{0.94\lambda }{{\beta \;1\text{/}2\;\cos \theta }}. $$

where λ is the wavelength (Cu-K), β_1/2_ is the broadening of the diffraction line measured at half of the maximum intensity, θ is the Bragg angle for a given diffraction, and Dp represents the crystallite size. The size of LNCN7 nanocomposite (14.30 nm) was slightly reduced compared with pure LaNiO_3_ (Dp = 16.23 nm) from the highest intensity peak of the (1 1 0) plane. Guessing that maybe because the aggregated LaNiO_3_ particles were greatly distributed on the surface of g-C_3_N_4_ nanosheets. All above these results confirmed the establishment of the interaction between LaNiO_3_ and g-C_3_N_4_.

### SEM and TEM analysis

**Fig. 3:**
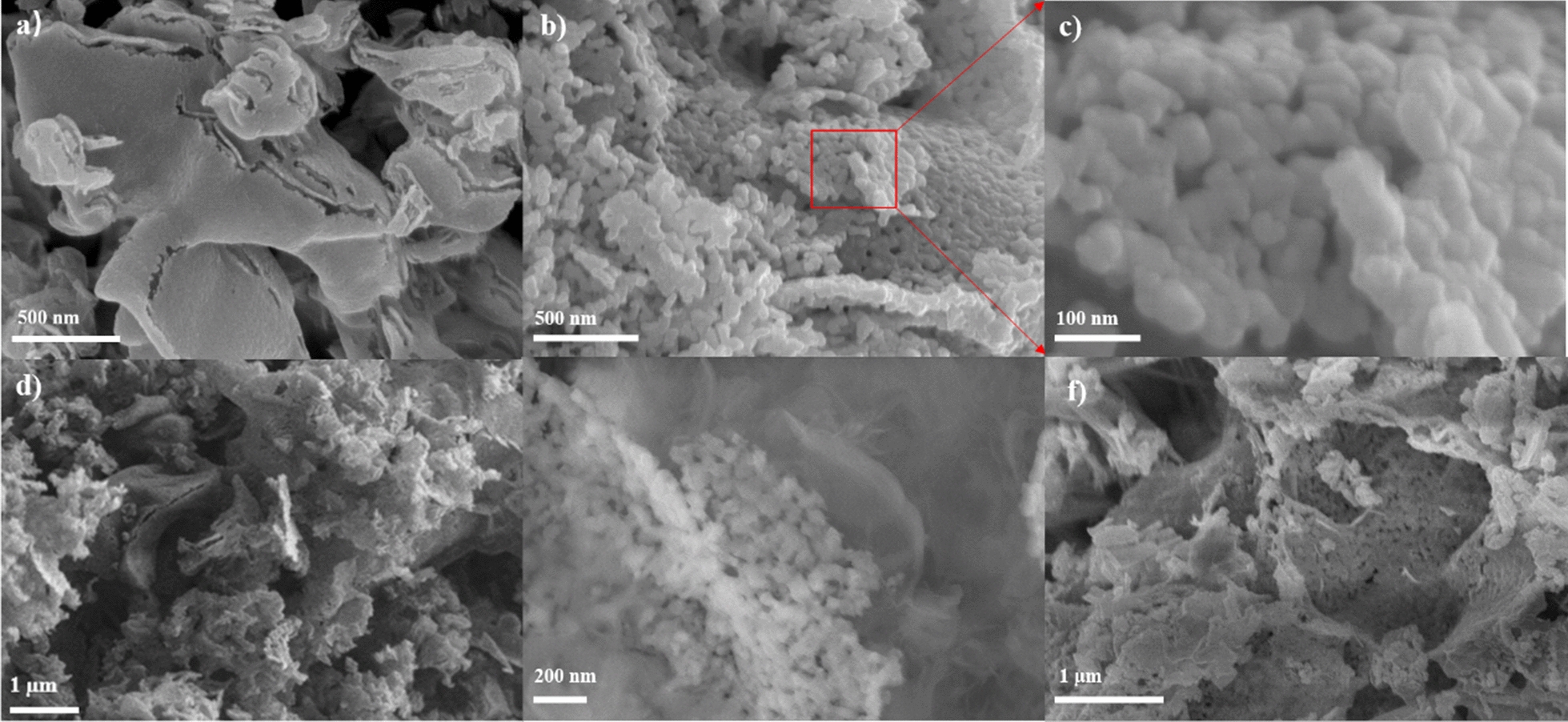
SEM images of **a** g-C_3_N_4_, **b, c** LaNiO_3_; **d, e** LaNiO_3_/70%g-C_3_N_4_; **f** LaNiO_3_/70%g-C_3_N_4_ after catalytic reaction

The surface microscopic morphologies of the g-C_3_N_4_, LaNiO_3_ and LNCN7 samples were tested by SEM. Figure 3(a) depicted that g-C_3_N_4_ were stacked by amount of irregular layered nanosheets structure and had relatively bigger smooth surface thus can be better as a substrate. From Fig. 3b, c, indicating the pure LaNiO_3_ were consisted of many aggregated spherical nanoparticles. The SEM images of Fig. 3d, e clearly revealed that LaNiO_3_ particles were well dispersed and deposed on the surface of g-C_3_N_4_. Figure 3(f) further present the morphology of LNCN7 after 20 h photocatalytic water splitting experiments, the picture showed that the catalyst still almost held the original appearance owing to its good stability and strong interaction on the interface.

**Fig. 4:**
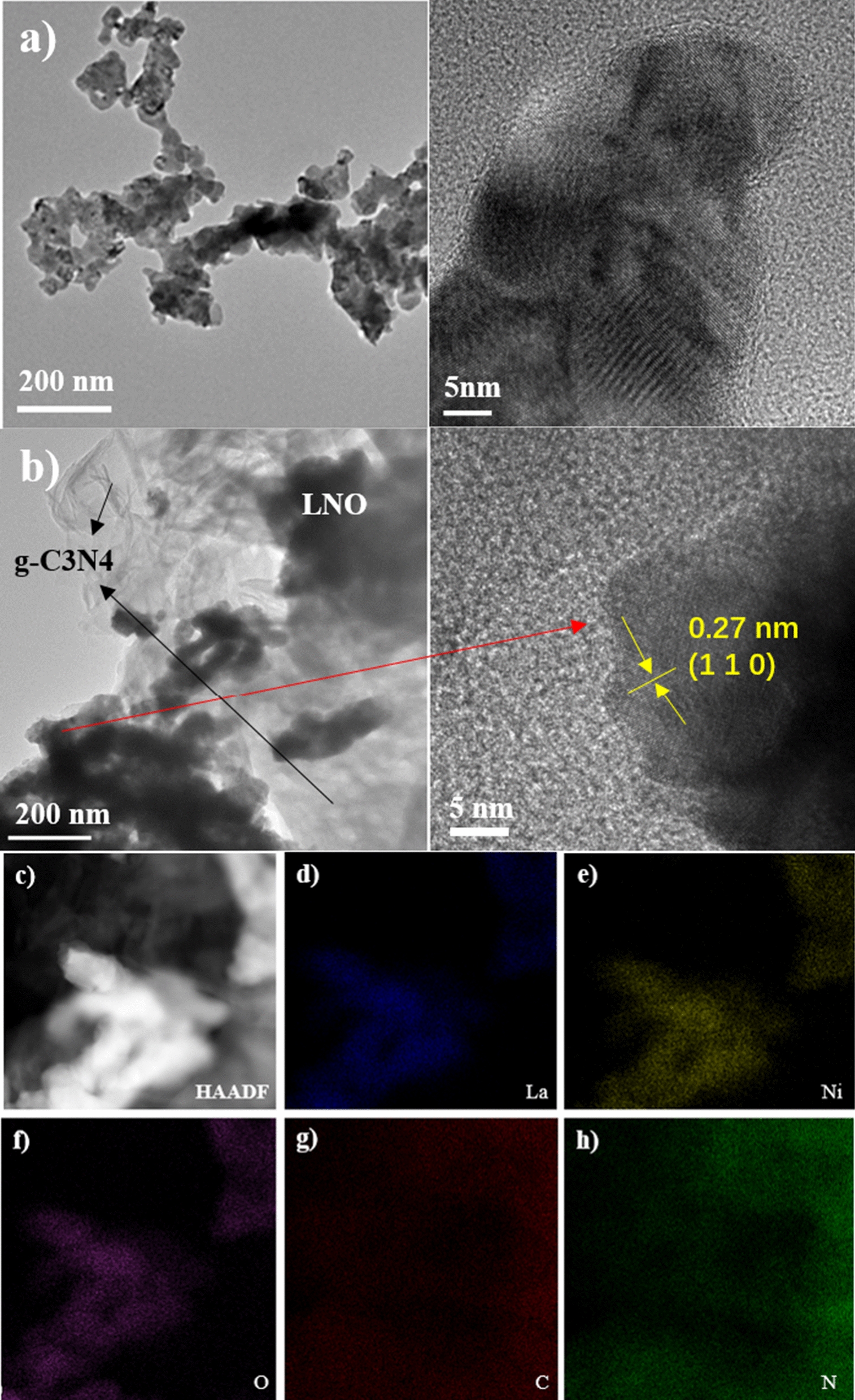
**a** TEM and HRTEM of LaNiO_3_; **b** TEM and HRTEM of LaNiO_3_/70%g-C_3_N_4_; **c** HAADF-STEM image and **d**–**h** elemental mappings of LaNiO_3_/70%g-C_3_N_4_

Moreover, the TEM and HRTEM analysis was further performed to verify the internal microstructure and nanoparticles size of as-obtained catalysts. Figure 4(a) shows the TEM and HRTEM of pristine LaNiO_3_, from the pictures, we could know the LaNiO_3_ sphere-like aggregated particles with a mean diameter about 20 nm which was basically consistent with the calculation result of crystallite size (Dp = 16.23 nm) obtained from XRD. The appropriately smaller particle size of our as-prepared perovskite-type semiconductor had several advantages for photocatalytic reaction. On the one hand, shortening the distance of photogenerated carriers migrated from the interior of the catalyst to the surface, thereby increasing the carriers’ migration rate and reducing the recombination rate. On the other hand, increasing the atomic number on the surface of particle and thus improving light absorption efficiency. Besides, smaller particle size nanoparticles have larger specific surface area and more reactive centers. Figure 4(b) displayed the TEM and partial area’s HRTEM images of Z-scheme heterostructure catalyst LNCN7 with a fringe width of about 0.27 nm indexed to the (1 1 0) plane of LaNiO_3_. It appeared that large amounts of dark aggregated particles were well dispersed on the big irregular wrinkled surface which could be considered that these LaNiO_3_ particles had successfully loaded on the surface of g-C_3_N_4_ and formed the heterostructure. This structure would be beneficial to enhance the photocatalytic performance by improving the separation efficiency of electrons and holes. Besides, corresponding to the highest intensity (1 1 0) plane of LaNiO_3_ crystals, we clearly observed lattice fringes with a width of about 0.27 nm. Figure 4(c–h) presented the high-angle annular dark-field scanning transmission electron microscopy (HAADF-STEM) and corresponding elemental mapping images of selected area of LNCN7, from which we could observe the composition and distribution of elements on this composite. It can be obtained that both C and N elements were distributed throughout the images (Fig. 4g, h) and La, Ni, O three kinds elements were exactly situated at the location of LaNiO_3_ particles (Fig. 3d–f). Additionally, from the energy dispersive X-ray (EDX) spectra in Additional file 1: Fig. S1, we would once again confirm the formation of LaNiO_3_/g-C_3_N_4_ and this result would be further supported by XPS analysis.

### XPS analysis

**Fig. 5:**
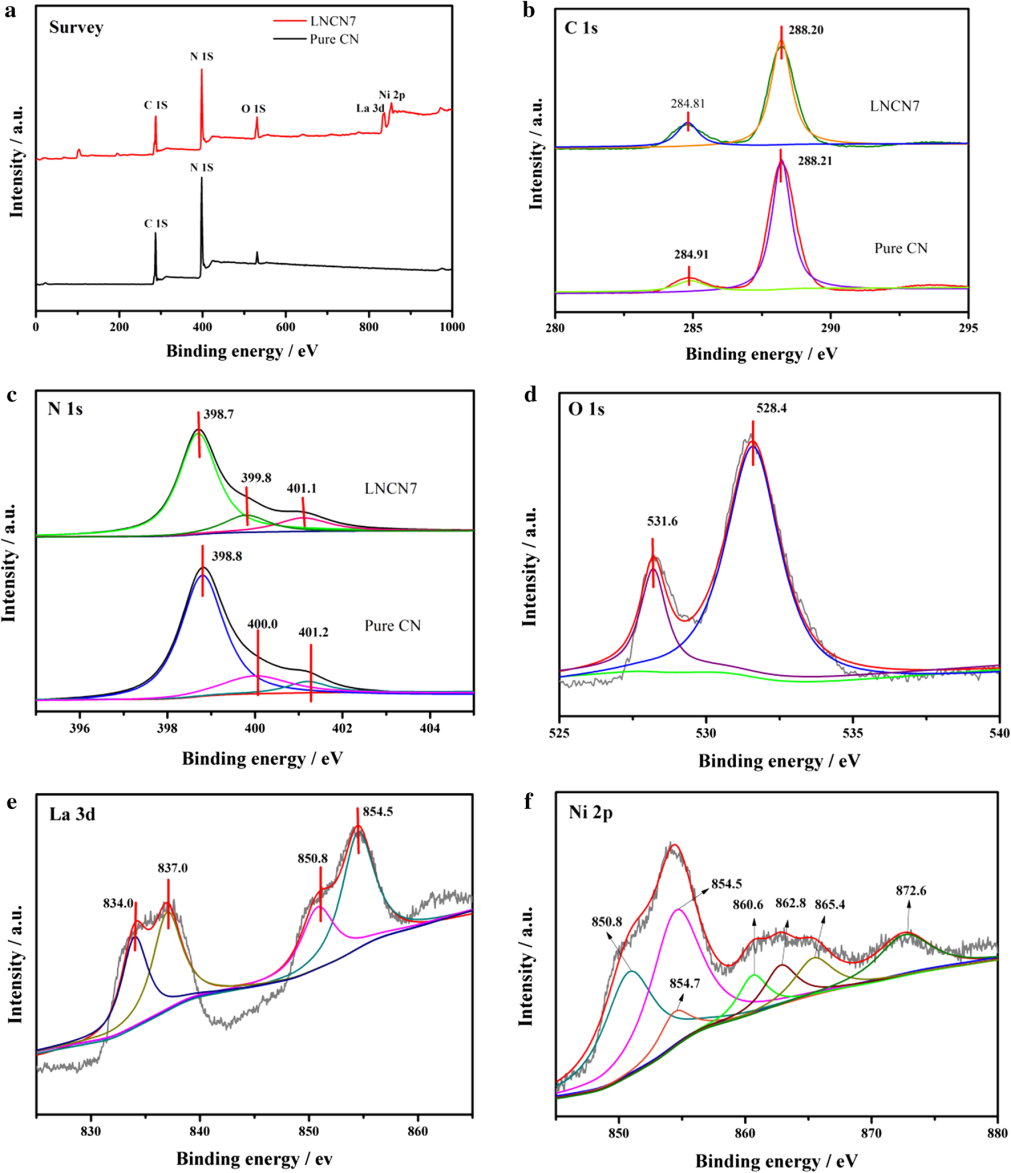
**a** XPS spectrum surveys scan for LNCN7 and pure CN; high resolution **(b)** C 1 s and (**c**) N 1 s for LNCN7 and pure CN; high resolution XPS spectra of (**d**) O 1 s, **e** La 3d and **f** Ni 2p for LNCN7 composite

As displayed in Fig. 5 X-ray, photoelectron spectroscopy (XPS) tests were conducted to verify the surface chemical composition and the chemical states for the LNCN7 sample. Figure 5a depicted the overall XPS survey spectrum, it revealed that C and N elements were detected in both pristine g-C_3_N_4_ and LaNiO_3_/70%g-C_3_N_4_, while elemental signals of La, Ni and O were only observed in the latter, which were well consistent with the results of EDX measurements. High resolution XPS spectra, as performed in Fig. 5(b–f), were further analyzed to obtain the information of chemical states. In the high resolution C 1 s XPS spectra (Fig. 5b), two main distinctly peaks at 284.91 eV and 288.21 eV were observed for pure g-C_3_N_4_, which were indexed to sp^2^ C–C bond and N–C = N bond in the aromatic ring of g-C_3_N_4_ [49]. For LNCN7, the binding energies of C 1 s at 284.81 eV and 288.20 eV shifted to lower binding energies than pure g-C_3_N_4_. In Fig. 5(c), three main peaks for N 1 s of g-C_3_N_4_ were observed at 398.8 eV, 400.0 eV and 401.2 eV, these peaks could be attributed to the N = C-N, N-(C)_3_ and C-N–H, respectively [50, 51]. Surprisingly, which was similar to C 1 s, the binding energies of N 1 s for LNCN7 sample also shifted to lower binding energies (398.7, 399.8, 401.1 eV) by about 0.1–0.2 eV compared with g-C_3_N_4_. The similar phenomenon might result from the formation and presence of the strong interaction between LaNiO_3_ and g-C_3_N_4_, which could demonstrate that the chemical states of C and N surroundings in LNCN7 had changed [52]. In the case of high resolution O 1 s spectra (Fig. 5d) for LNCN7, the peak could be deconvoluted into two main peaks at 531.6 eV and 528.4 eV, corresponding to the surface adsorbed oxygen and the crystal lattice oxygen. Figure 5e showed the high resolution spectrum of La 3d for LNCN7, four peaks at 834.0, 837.0, 850.8, and 854.5 eV were observed to correspond to the binding energies of La 3d_5/2_ and 3d_3/2_, respectively. Importantly, it could confirm the existence of La^3+^ ions in oxide [52, 53]. As shown in Fig. 5f, two La 3d_3/2_ peaks (850.8 and 854.5 eV) were overlapped with peaks of nickel. Besides, the peaks at 854.7, 860.6, 862.8, 865.4, and 872.6 eV were indexed to the binding energies of Ni 2p_3/2_ and Ni 2p_1/2_ for LNCN7 composite, which was characteristic of the Ni^3+^ cation [54]. Above all, these XPS results further confirmed that the successful synthesis of LaNiO_3_/g-C_3_N_4_ heterojunction nanocomposites.

### FTIR spectra

**Fig. 6:**
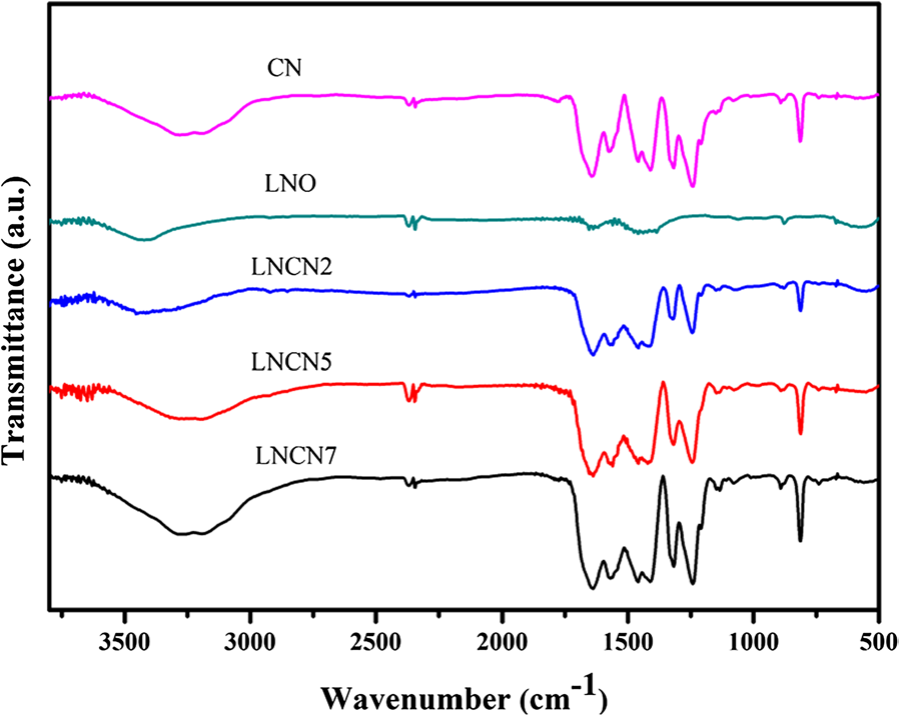
FTIR spectra of g-C_3_N_4_, LaNiO_3_ and LaNiO_3_/g-C_3_N_4_

The Fourier transform infrared (FTIR) spectra of as-obtained g-C_3_N_4_, LaNiO_3_ and a series of LaNiO_3_/g-C_3_N_4_ were displayed in Fig. 6, which present the change of different spectra along with the progressively increase of CN in LNCN composite. In the spectra of g-C_3_N_4_ and LaNiO_3_/g-C_3_N_4_, the obviously absorption peak nearly at 3200 cm^−1^ was regarded as the stretching and bending vibrations of N–H which come from the uncondensed terminal amino groups. This indicates that the amino functions still existed in the products by directly heating the urea. In the region of 1200–1650 cm^−1^, several strong peaks were ascribed to typical aromatic heterocycle stretches of the g-C_3_N_4_ [55, 56]. For pure g-C_3_N_4_, the characteristic peaks at 1294.5, 1435.6, and 1597.2 cm^−1^ were consistent with the stretching modes of the C-N framework structure of the conjugated aromatic ring. Moreover, the breathing vibration of s-triazine for g-C_3_N_4_ result in the characteristic absorption peak at 810 cm^−1^ [57]. What’s more, the characteristic peaks of LaNiO_3_ at 565 cm^−1^ were related to the bending and tensile vibration of Ni–O and correspond to the perovskite structure. Strikingly, accompanying with the increase of g-C_3_N_4_ content in LNCN samples, the intensities of above three types of peaks were gradually strengthened, which may be due to the fact that the formation of heterojunction changes the chemical environment around the chemical bond.

### BET analysis

**Fig. 7:**
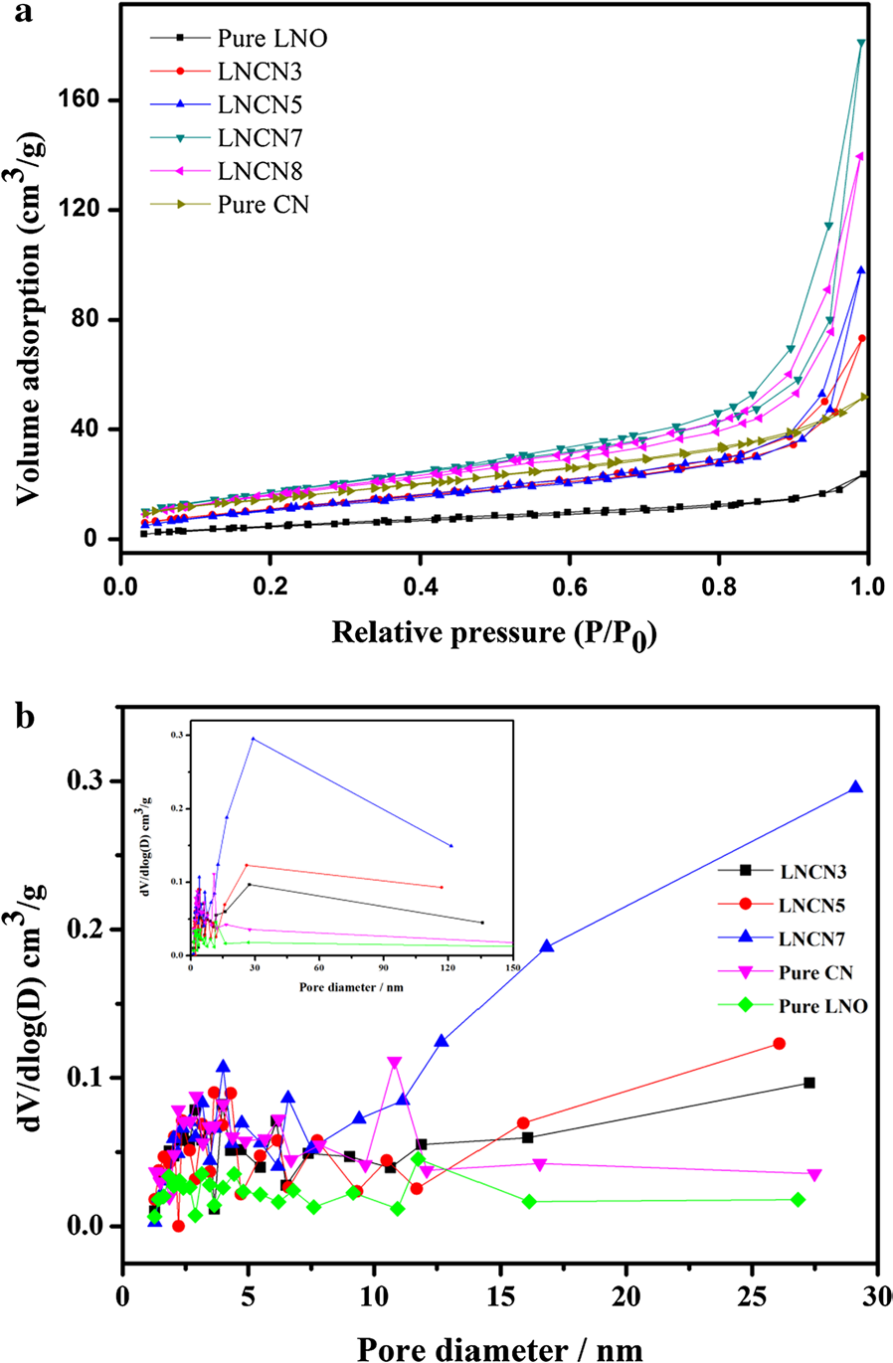
Nitrogen adsorption–desorption isotherms (**a**) and pore size distribution curves; **b** of all as-obtained samples

The porous structures and specific surface areas of as-obtained samples were characteristic using nitrogen adsorption–desorption experiments at 77 K, and the corresponding curves and data were shown in Fig. 7 and Additional file 1: Table S1. As shown in Fig. 7a, we could see that all as-obtained samples reveal type IV curves with an H3-type hysteresis loop, according to the Brunauer-Deming-Deming-Teller (BDDT) classification, which represent that the existence of abundant mesoporous structure in our samples [45]. Figure 7b further depicted the corresponding BJH pore size distribution curves, suggesting that these samples had a wide pore size range of 2–120 nm and most of the pore diameters were in the range of 2-12 nm. This figure further illustrated that the material we prepared own mesoporous structure. From the figure we could see that the pore volume of LNCN7, LNCN5, LNCN3, pure CN and pure LNO showing a decreasing pattern. Mesoporous structure could provide more reaction sites and active sites so that strengthen photocatalytic activity and accelerate the reaction rate.

What’s more, Additional file 1: Table S1 clearly displayed the Brunauer–Emmett–Teller (BET) specific surface areas, mean pore diameter and pore volume of pure g-C_3_N_4_, LaNiO_3_ and LaNiO_3_/g-C_3_N_4_ composites. The mean pore diameter listed in the table further demonstrated that our as-obtained samples were classified to mesoporous structure materials. Evidently, the BET specific surface areas of LNCN composites were gradually larger than that of pristine LaNiO_3_ with the rising of content, and then slightly decreased with the further increasing of g-C_3_N_4_ content. As we all know, normally, the larger specific surface area also could improve light absorption efficiency and provide more reaction sites so that strengthen photocatalytic activity and accelerate the reaction rate. Coincidently, the pore volume also exist the similar trend of changing. From the Additional file 1: Table S1 we could observed that the LaNiO_3_/70%g-C_3_N_4_ composites gained the highest BET specific surface area (66.06 m^2^ g^−1^) and the maximum pore volume (0.289 cc g^−1^) compared with others. In the following part, the photocatalytic experiment would also indicated that LNCN7 sample had the fastest hydrogen evolution rate, which proved that the BET specific surface area and pore volume might performed extremely important characters for photocatalytic properties. So what caused the changing of specific surface area? After the formation of the heterojunction material, the originally aggregated LaNiO_3_ nanoparticles were uniformly dispersed on the g-C_3_N_4_ nanosheets and thus exposed a larger reaction surface. When the doping ratio of g-C_3_N_4_ achieved 70%, nanoparticles could exactly uniformly distribute on the nanosheets and achieve the optimal doping state.

### Photocatalytic activity and stability for H_2_ production

**Fig. 8:**
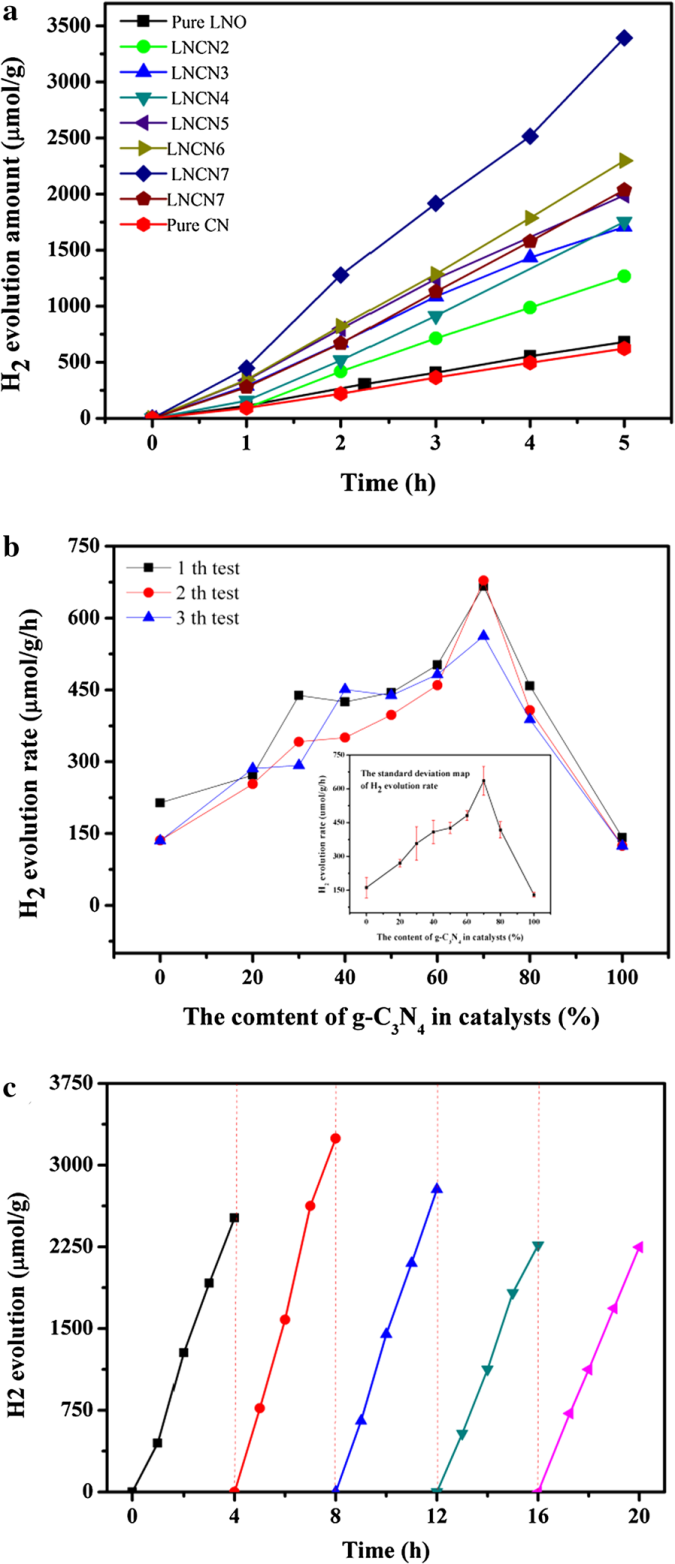
**a** Photocatalytic H_2_ evolution rate as a function of irradiation time of 5 h over LNO, CN and LNCN [2–8]; **b** Photocatalytic H_2_ evolution rate for three repeated tests over samples with different content of g-C_3_N_4_ and the corresponding standard deviation map; **c** Long-term stability test of LNCN7 composite for 20 h

Photocatalytic hydrogen evolution performances of synthesized LNO, CN, and LNCN nanoparticles were tested in aqueous solution containing 10 vol % methanol as sacrificial reagent under 300 W Xe lamp irradiation which was equipped with a filter with a wavenumber of 250–380 nm. The H_2_ evolution amount shown in Fig. 8 was detected by means of a gas chromatograph. As shown in Fig. 8a, b, we performed three parallel tests over all samples and calculated the average value of H_2_ evolution rate so that we could eliminate the accidental factors and obtain more convincing data. The H_2_ evolution rate for neat LaNiO_3_ and g-C_3_N_4_ was 135.9 µmol h^−1^ g^−1^ and 124.4 µmol h^−1^ g^−1^, respectively. Obviously, the LNCN heterojunction composites universally showed higher H_2_ evolution activity compared with neat LNO and CN. In all composite materials, the H_2_ evolution rate gradually increased with the increasing of g-C_3_N_4_ content. LNCN7 composite reached an optimal yield of up to 3392.50 µmol g^−1^ in 5 h and possessed the highest rate of 678.5 µmol h^−1^ g^−1^ which was nearly 5 times than that of pure LNO. The improvement of photocatalytic activity may be due to the attendance of g-C_3_N_4_ would reduce the recombination rate of photoexcited carriers effectively and generate more effective electrons in LNCN Z-scheme system for generating hydrogen. Besides, the doping ratio could change the distribution state of nanoparticles on the nanosheet, and then affect the material’s crystal structure, specific surface area, pore size, pore volume and other properties, thereby affecting the catalytic activity. A series of characterization tests revealed that heterojunction catalyst with the content of g-C_3_N_4_ in LNCN was 70% present the superior crystal phase structure, the largest specific surface area and pore volume. These features were beneficial to enhance the activity of catalytic hydrogen production. On the contrary, further improving the content of g-C_3_N_4_ would reduce the catalytic effect. Therefore, LNCN7 has the largest hydrogen production rate.

As presented in Fig. 8c, the long-term photocatalytic stability experiment of LNCN7 was conducted for five cycles under same reaction conditions. It can be seen that the H_2_ production amounts of the fifth cycle for LNCN7 was reduced to 2246.9 µmol g^−1^ after 5 h and only 10.7% H_2_ production was lost compared with the first cycle. The result revealed that LNCN7 composite almost held stable during the long-term test and it was provided with good stability in the photocatalytic reaction.

**Fig. 9:**
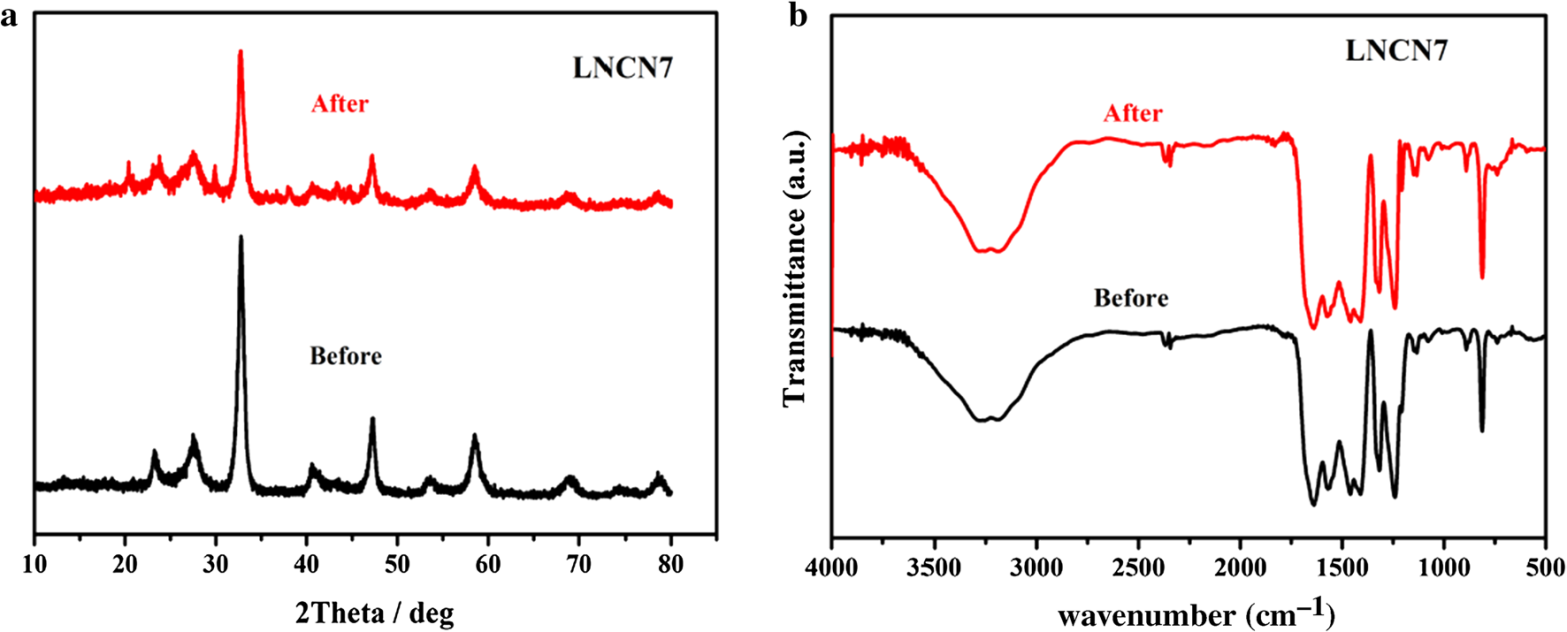
XRD diffraction patterns (**a**) and FT-IR images (**b**) of LNCN7 before and after long-term photocatalytic experiment

What’s more, there was no distinct change in the XRD diffraction patterns and FT-IR images of LNCN7 before and after five cyclic photocatalytic experiments, as observed in Fig. 9a, b, respectively. The results further confirmed the former conclusion that our synthesized LNCN7 nanoparticles had good stability and sturdy crystal structure.

### Proposed photocatalytic mechanism for H_2_ production

**Fig. 10:**
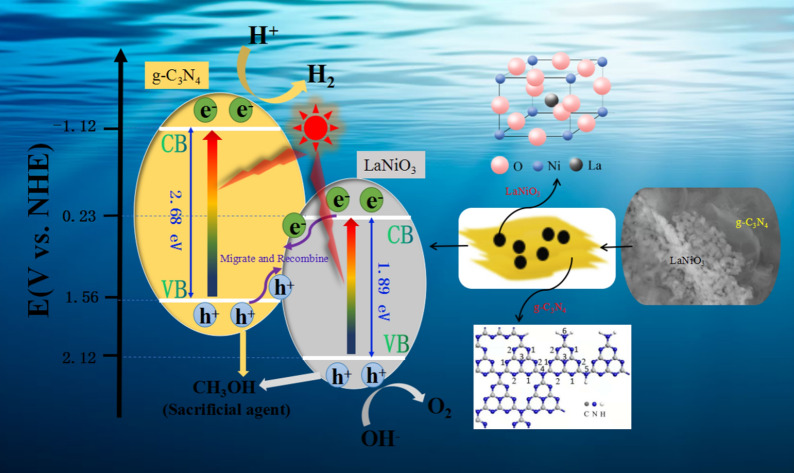
The proposed photocatalytic H_2_ production mechanism using CH_3_OH as the sacrificial agent over LaNiO_3_/g-C_3_N_4_ heterostructure nanocomposites

On the basis of above analysis and experimental results, LaNiO_3_/g-C_3_N_4_ heterojunction nanocomposite performed higher H_2_ evolution efficiency than that of pure LaNiO_3_ and g-C_3_N_4_, which might result from the combination of these two semiconductors and the formation of the solid–solid interfacial between them. As depicted in Fig. 10, a direct Z-scheme photocatalytic mechanism was proposed to specifically describe the migration route of photogenerated carriers and the related reactions process in the system [58, 59]. We could utilize the energy band theory of semiconductors to explain the principle of photocatalytic hydrogen production reaction. All semiconductors existed the forbidden band width, and they were excited to generate electron–hole pairs under light irradiation when the photon energy was equal or higher than the width of forbidden band. Because of owing the narrow band gap, electrons in both LaNiO_3_ and g-C_3_N_4_ were excited to transfer from valance band (VB) to conduction band (CB) under UV-light irradiation, and thus took shape the electron–hole pairs.

Here, existing two typical photogenerated charge carrier separation mechanism for the binary heterojunction system: the traditional transfer mechanism and the direct Z-scheme mechanism [52]. We assumed that the system made use of the former type of mechanism. In this situation, the photogenerated electrons in the CB of g-C_3_N_4_ would migrate to that of LaNiO_3_, which could weaken the reducibility of photoexcited electrons (potential energy from −1.12 eV to 0.23 eV). And the electrons gathered in the CB of LNO didn’t have the capability to reduce H^+^ into H_2_ due to the more negative standard reduction potential of H_2_O/H_2_ (−0.42 eV). Similarly, the holes generated in the VB of LNO also moved to that of g-C_3_N_4_, which would lead to the decline of oxidizing ability. Therefore, the traditional transfer mechanism was not applicable to our photocatalytic system. As a result, the charge transfered in LNCN most likely followed by a Z-scheme mechanism. Under the electrostatic interaction of the material interior, the photogenerated electrons from the CB of LaNiO_3_ and the photogenerated holes from the VB of g-C_3_N_4_ individually migrated to the solid–solid hetero-interfacial and then recombined. The recombination behavior improved the separation of the photogenerated carriers both in LaNiO_3_ and g-C_3_N_4_, effectively. The electrons accumulated in the CB of g-C_3_N_4_ participated in H_2_ evolution reaction and thus we could get the target product. Simultaneously, the extra holes from the VB of LaNiO_3_ and g-C_3_N_4_ were captured and depleted by the sacrificial agent (methanol) in order to enhance the photocatalytic effect. To sum up, the specific reaction process could refer as following steps:

2 $$ \text{LaNiO}_{\text{3}} \;\text{/}\;\text{g} - \;\text{C}_{\text{3}} \text{N}_{\text{4}} \;{ + hv \to e}_{{\left( {\text{LNO}} \right)}}^ - \;\text{ + }\;\text{h}_{{\left( {\text{LNO}} \right)}}^{\text{ + }} \;\text{ + }\;\text{e}_{{\left( {\text{CN}} \right)}}^ - \;\text{ + }\;\text{h}_{{\left( {\text{CN}} \right)}}^{\text{ + }} $$

3 $$ \text{e}_{{\left( {\text{LNO}} \right)}}^ -  \;\text{ + }\;\text{h}_{{\left( {\text{CN}} \right)}}^{\text{ + }} \;{ \to }\;\text{Migration}\;\text{and}\;\text{recombination} $$

4 $$ \text{H}_{\text{2}} \text{O}\;\text{ + }\;\text{2h}_{{\left( {\text{LNO}} \right)}}^{\text{ + }} \;{ \to }\;\text{1/}\;\text{2O}_{\text{2}} \;\text{ + }\;\text{2H}^{\text{ + }} $$

5 $$ 2e_{{\left( {CN} \right)}}^ -  \;\text{ + }\;2H^{\text{ + }} \; \to \;H_{2} \;\left( g \right) $$

## Conclusions

In this study, a direct Z-scheme heterostructure photocatalyst LaNiO_3_/g-C_3_N_4_ with different content of g-C_3_N_4_ was successfully fabricated through a facile ultrasonication strategy, continuous stirring process and solvothermal method, successively. We already confirmed that the as-obtained LNCN nanoparticles formed strong interaction between the solid–solid interface of LaNiO_3_ and g-C_3_N_4_ by means of a series of characterization methods. No surprisingly, all as-prepared LNCN samples universally exhibited enhanced performance towards photocatalytic water splitting reaction under UV-light irradiation even with the absence of co-catalyst like noble metal. It was worth mentioning that LaNiO_3_/70%g-C_3_N_4_ composite displayed the highest H_2_ evolution rate of 678.5 µmol h^−1^ g^−1^ which was almost 5 times than that of single LaNiO_3_ (135.9 µmolh^−1^g^−1^). Besides, the LNCN7 heterojunction nanocomposite behaved great photocatalytic stability during the long-term reaction process. The effective improving of hydrogen production rate for LNCN composites might result from the strong interfacial interaction which could electrons from LNO and holes from CN migrated and recombined, and thus suppress the recombination of photogenerated charge carriers both in LNO and CN. As a result, the LNCN Z-scheme heterostructure photocatalysis system would not only broaden the light response area and promote the utilization of solar energy, but also enhance the photocatalytic ability. In conclusion, facing the situation of environmental remediation and solar energy conversion, it was worth taking further actions to explore and develop more novel Z-scheme photocatalysts based on perovskite or polymer which were highly-efficient, economical and environmentally friendly.

## Supplementary information

**Additional file 1: Figure S1.** The HAADF image and EDX spectra of LaNiO_3_/70%g-C_3_N_4_. **Table S1.** The BET specific surface area, mean pore diameter and pore volume of the as-obtained samples

## Data Availability

All data generated or analysed during this study are included in this published article.
