# Supplementary material for: The high photocatalytic efficiency and stability of LaNiO3/g-C3N4 heterojunction nanocomposites for photocatalytic water splitting to hydrogen
Source: BMC Chem. 2020 Oct 29;14(1):65. doi: 10.1186/s13065-020-00719-w (PMC7596961; doi:10.1186/s13065-020-00719-w)
Supplement: Supplementary file 1 — Additional file 1: Figure S1. The HAADF image and EDX spectra of LaNiO3/70%g-C3N4. Table S1. The BET specific surface area, mean pore diameter and pore volume of the as-obtained samples [file 13065_2020_719_MOESM1_ESM.docx]

Fig. S1. The HAADF image and EDX spectra of LaNiO_3_/70%g-C_3_N_4_


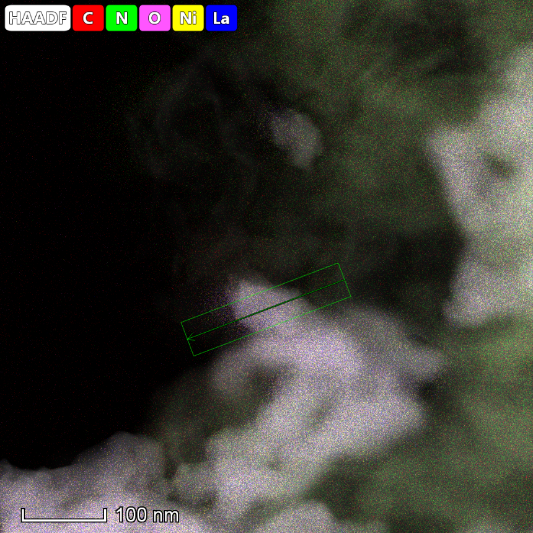

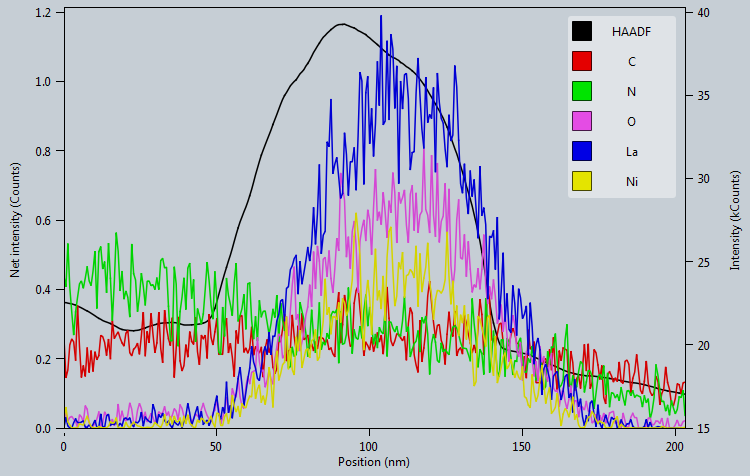


Table. S1. The BET specific surface area, mean pore diameter and pore

volume of the as-obtained samples.

| Samples | *S_BET_* (m^2^/g) | *V_total_* (cc/g) | *D_BJH_* (nm) |
| --- | --- | --- | --- |
| g-C_3_N_4_ | 54.352 | 0.084 | 2.212 |
| LaNiO_3_ | 21.277 | 0.042 | 1.839 |
| LaNiO_3_/(30wt%)g-C_3_N_4_ | 43.033 | 0.121 | 1.853 |
| LaNiO_3_/(40wt%)g-C_3_N_4_ | 40.111 | 0.172 | 2.190 |
| LaNiO_3_/(50wt%)g-C_3_N_4_  LaNiO_3_/(60wt%)g-C_3_N_4_  LaNiO_3_/(70wt%)g-C_3_N_4_  LaNiO_3_/(80wt%)g-C_3_N_4_ | 43.223  48.261  66.060  62.377 | 0.163  0.187  0.289  0.226 | 2.366  2.184  2.027  2.201 |

*S_BET_* is the speciﬁc surface area calculated from the N_2_ adsorption isotherm according to the BET method. *V_total_* is the single-point total pore volume. *D_BJH_* is the pore size determined from the N_2_ desorption branch using the BJH model.
